# Supplementary material for: Effect of Lactic Acid Fermentation on Color, Phenolic Compounds and Antioxidant Activity in African Nightshade
Source: Microorganisms. 2020 Aug 30;8(9):1324. doi: 10.3390/microorganisms8091324 (PMC7564239; doi:10.3390/microorganisms8091324)
Supplement: Supplementary file 1 [file microorganisms-08-01324-s001.pdf]

Supplementary Table 1 Pearson's correlation coefficients of phenolic profiles and antioxidant (FRAP) activities

	Correlation co-efficient
	FRAP
Total polyphenols	**0.82**
Gallic acid	0.645
Catechin.	0.541
Caffeic acid	-0.343
Vanillic	**0.726**
2.4 Dihydroxybenzoic acid	-0.134
Coumaric	**0.868**
Ferulic	0.459
Ellagic	-0.842
Quercetin	0.091
Luteolin	0.510

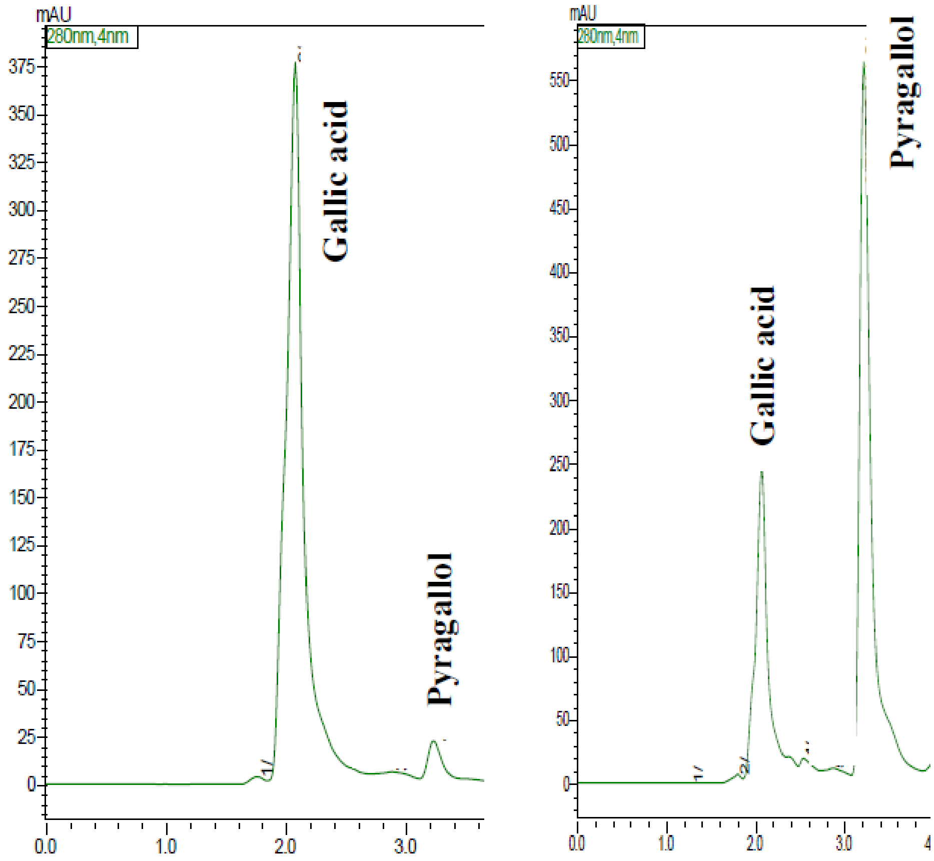

Supplementary Fig. 1 Chromatogram showing the changes in gallic acid concentration and pyragallol during fermentation by *Weissella cibaria* (strain *21*) and *Lactobacillus plantarum* (strain *75*)
